# Clinical evaluation of personalized *Helicobacter pylori* treatment guided by PCR detection from fecal samples: a real-world study

**DOI:** 10.3389/fcimb.2025.1519804

**Published:** 2025-04-28

**Authors:** Ruolin Peng, Zhimei Zhang, Chuchu Yang, Zhengyuan Xu, Jiabin Wang, Lei Chen, Sujun Gao, Jian Tao, Meijuan Xi, Xiaofeng Ye, Lu Shen, Qiang Zhan, Lijia Din, Jun Wang, Rong Ou, Jianhua Cui, Lin Chen, Xiaodan Xu, Gongyu Zhang, Chunyan Xu, Jing Du, Guangxia Chen, Xinxin Zhao, Lamei Xu, Min Xu, Zhenyu Zhang

**Affiliations:** ^1^ Department of Gastroenterology, Nanjing First Hospital, Nanjing Medical University, Nanjing, Jiangsu, China; ^2^ Department of Gastroenterology, Lianyungang Clinical College of Nanjing Medical University, Lianyungang, Jiangsu, China; ^3^ Department of Gastroenterology, The Affiliated Shuyang Hospital of Xuzhou Medical University, Suqian, Jiangsu, China; ^4^ Department of Gastroenterology, Northern Jiangsu People’s Hospital, Yangzhou, Jiangsu, China; ^5^ Department of Gastroenterology, Changshu Hospital of Traditional Chinese Medicine, Suzhou, Jiangsu, China; ^6^ Department of Gastroenterology, Changzhou Traditional Chinese Medicine Hospital, Changzhou, Jiangsu, China; ^7^ Department of Gastroenterology, The Affiliated Wuxi People’s Hospital of Nanjing Medical University, Wuxi, Jiangsu, China; ^8^ Department of Gastroenterology, Jinhu County People’s Hospital, Huaian, Jiangsu, China; ^9^ Department of Gastroenterology, Dongtai People’s Hospital, Yancheng, Jiangsu, China; ^10^ Department of Gastroenterology, Changshu No.1 People’s Hospital, Suzhou, Jiangsu, China; ^11^ Department of Gastroenterology, The Affiliated Huaian Hospital of Yangzhou University, The Fifth People’s Hospital of Huaian, Huaian, Jiangsu, China; ^12^ Department of Gastroenterology, The First People’s Hospital of Xuzhou, Xuzhou Municipal Hospital Affiliated to Xuzhou Medical University, Xuzhou, Jiangsu, China; ^13^ Department of Gastroenterology, Affiliated Hospital of Jiangsu University, Zhenjiang, Jiangsu, China

**Keywords:** *Helicobacter pylori*, stool, PCR, drug resistance, personalized eradication

## Abstract

**Background:**

Growing antibiotic resistance in *Helicobacter pylori* (*H. pylori*) diminishes eradication therapy effectiveness, emphasizing the need for accurate, non-invasive diagnostic techniques. This study aims to assess the clinical utility of PCR analysis of fecal samples for detecting antibiotic resistance in guiding personalized treatment for *H. pylori* infection.

**Methods:**

A retrospective, observational study was conducted across 13 hospitals within Jiangsu Province. Fecal samples were analyzed using fluorescence PCR for the *23S rRNA* and *gyrA* genes, indicating clarithromycin and levofloxacin resistance. Then, individualized eradication recommendations were proposed for the *H. pylori*-positive patients. A follow-up was performed one year later to evaluate the eradication outcomes in a routine medical environment, with participants having provided informed consent.

**Results:**

A total of 387 participants completed the eradication treatment, with an overall success rate of 91.0% (352/387, 95% CI: 87.6%-93.6%). Among them, 310 individuals received a 14-day course of bismuth quadruple therapy (BQT), achieving an eradication rate of 90.0% (279/310, 95% CI: 86.1%-93.1%). 77 participants were treated with high-dose dual therapy (HDDT) for the same duration, resulting in a slightly higher eradication rate of 94.8% (73/77, 95% CI: 87.2%-98.6%), although this difference was not statistically significant (*P*=0.188). No significant differences in eradication rates were observed among various BQT antibiotic combinations (*P*=0.208). The eradication rates for HDDT, based on either vonoprazan or esomeprazole, were 96.8% (61/63, 95% CI: 89.0%-99.6%) and 85.7% (12/14, 95% CI: 57.2%-98.2%), respectively, without a significant difference (*P*=0.304).

**Conclusion:**

PCR detection from fecal samples targeting the resistance genes of *H. pylori* is effective in guiding personalized treatments, highlighting its clinical utility and potential for broader application.

## 1 Introduction

In 1994, the World Health Organization classified *Helicobacter pylori* (*H. pylori*) as a Group I carcinogen for gastric cancer (Moller et al., 1995), and nearly 89% of gastric cancer cases worldwide are caused by *H. pylori* infection (Malfertheiner et al., 2022). The global prevalence of *H. pylori* is estimated at approximately 48.9%, despite the fact that there is a declining trend (Li et al., 2023). It is widely recognized that *H. pylori*-positive patients should undergo eradication therapy in the absence of contraindications or resistance factors (Sugano et al., 2015). However, the increasing resistance of *H. pylori* to antibiotics has led to unsatisfactory eradication rates. The selection of sensitive antibiotics is a primary determinant of the success of *H. pylori* eradication efforts (Liou et al., 2020).

Traditional antimicrobial susceptibility testing for *H. pylori* continues to rely on the laborious and time-consuming process of bacterial culture. Molecular biology techniques have emerged as an alternative approach, offering rapid, accurate diagnosis of *H. pylori* antibiotic resistance through the detection of specific genotypes. At present, the *23S rRNA* and *gyrA* genes are recognized as reliable markers for predicting resistance to clarithromycin and levofloxacin, respectively. However, for other antibiotics, the genotypes associated with resistance remain poorly defined (Zhong et al., 2021). Polymerase chain reaction (PCR) is a widely utilized molecular biology technique, recommended for the detection of clarithromycin and levofloxacin resistance genotypes in *H. pylori* (Malfertheiner et al., 2022). PCR has demonstrated sensitivities and specificities exceeding 95% when identifying *H. pylori* genotypes resistant to levofloxacin and clarithromycin in gastric fluid (Peng et al., 2017) and gastric mucosal samples (Li et al., 2020; Zhao et al., 2022).

Fecal PCR, in particular, has gained traction as a non-invasive and patient-friendly alternative. Unlike gastroscopy-dependent methods, fecal sampling eliminates procedural risks, reduces costs, and improves accessibility. Furthermore, stool-based DNA remains stable during intestinal transit, preserving target genotypes for reliable detection. The gastric mucosa is renewed approximately every three days, and *H. pylori*, along with the shed mucosa, can be excreted into the feces. Studies have shown that the clarithromycin and levofloxacin resistance genotypes detected in fresh gastric mucosal samples are strongly correlated with those identified in stool samples (Moss et al., 2022). Fan et al (Fan et al., 2024) reported that the specificity of real-time PCR for detecting resistance to clarithromycin and levofloxacin in fecal samples was 98.4% and 91.1%, respectively, compared to molecular detection from gastric biopsy samples. In summary, fecal PCR enables rapid and accurate diagnosis of *H. pylori* resistance to clarithromycin and levofloxacin through non-invasive sampling, thus serving as a scalable platform for large-scale resistance surveillance and personalized treatment strategies.

Multiple empirical studies have shown that fecal PCR testing for clarithromycin resistance facilitates effective eradication rates (Beckman et al., 2017; Marrero et al., 2021). Specifically, the correlation between PCR-based genotype prediction and the eradication of infection among patients treated with clarithromycin reached 83.5% (Beckman et al., 2017). Nevertheless, current evidence regarding the applicability of fecal PCR-guided personalized therapy in diverse clinical populations remains limited. While preliminary studies suggest promising eradication rates in controlled settings, the effectiveness of this approach across heterogeneous patient groups with varying comorbidities and adherence patterns requires further investigation. Through rigorous multicenter validation, this study seeks to contribute empirical data that may help refine implementation strategies, ultimately supporting more equitable access to precision *H. pylori* management.

## 2 Materials and methods

### 2.1 Study design

This retrospective, observational, and multicenter real-world study was conducted in compliance with the ethical principles stated in the Declaration of Helsinki. It received approval from the Medical Ethics Committee of the Nanjing First Hospital (approval number KY20221124-09).

### 2.2 *H. pylori* antibiotic resistance detection

Jiangsu Cowin Biotech Co., Ltd. provided technical support. The procedures were carried out in accordance with the instructions. The *23S rRNA* and *gyrA* mutation genotypes were detected to determine clarithromycin and levofloxacin resistance using the *H. pylori 23S rRNA*/*gyrA* gene mutation detection kit, which utilized the PCR melting curve method. PCR primers and mutation sites are shown in **Supplementary Table 1**. The PCR system consisted of 29 μL PCR premixes, 1 μL DNA polymerase, and 20 μL extracted sample DNA. PCR premixes included primers and probes for the *23S rRNA*, *gyrA*, and ACTB genes; dNTPs; Mg2+ ions; and Tris-hydrochloric acid buffer solution. The reaction conditions are detailed in **Supplementary Table 2**.

The relationship between genotype and drug resistance was categorized as follows: Samples with the *23S rRNA*/*gyrA* wild-type genotype were considered sensitive to clarithromycin and levofloxacin. Samples identified as the *23S rRNA*/*gyrA* mutant type were classified as resistant to both antibiotics. Furthermore, samples with the *23S rRNA*/*gyrA* mixed genotype were also rated as resistant to clarithromycin and levofloxacin.

### 2.3 Participants enrollment

The inclusion criteria required: (1) adults with confirmed *H. pylori* infection, (2) PCR-confirmed antibiotic resistance, and (3) voluntary participation. Participants were enrolled from 13 hospitals across Jiangsu Province between March 2023 and November 2023. Personalized eradication treatment recommendations were provided to *H. pylori*-infected individuals based on the test results. In a follow-up one year later, the treatment regimens received by these participants were assessed, and eradication outcomes were confirmed using urea breath test (UBT), without any intervention from the researchers.

### 2.4 Personalized treatment

Based on the PCR detection of *H. pylori* resistance to clarithromycin and levofloxacin in fecal samples, patients who tested positive for resistance to either antibiotic were advised to receive a bismuth quadruple therapy (BQT) or high-dose dual therapy (HDDT) that did not include the resistant antibiotics. For patients showing susceptibility to both antibiotics, there were no restrictions on the treatment. Treatment regimens, medication dosages, and durations were all in accordance with the 2022 Chinese national clinical practice guidelines for *H. pylori* eradication treatment. In brief, the BQT included 220 mg of bismuth and a standard dose of a proton pump inhibitor (PPI, such as rabeprazole 10 mg, omeprazole 20 mg, esomeprazole 20 mg, pantoprazole 5 mg, lansoprazole 40 mg, or lanzoprazole 30 mg), to be taken orally twice daily. Two of the following antibiotics were selected based on susceptibility results: amoxicillin 1000 mg BID, clarithromycin 500 mg BID, levofloxacin 500 mg QD, furazolidone 100 mg BID, tetracycline 500 mg TID or QID, and metronidazole 400 mg TID or QID. If tetracycline was not readily available, a semi-synthetic tetracycline could be substituted. The HDDT consisted of a double dose of a PPI (for example, esomeprazole 20 mg QID/40 mg BID or rabeprazole 10 mg QID/20 mg BID) combined with a high dose of amoxicillin (1000 mg TID/750 mg QID). Furthermore, if the new acid-suppressing agent vonoprazan (potassium-competitive acid blocker, P-CAB) was accessible, it could replace the PPI in the aforementioned regimens, with a recommended dosage of 20 mg BID. The recommended duration for all treatment plans was 14 days.

All patients went unassisted to the hospital to receive individualized *H. pylori* eradication treatment and follow-up, without any intervention from the researchers during the treatment process, ensuring the objectivity and authenticity of the study.

### 2.5 Statistical analysis

Data analysis was performed using SPSS software, version 25.0. Categorical data were expressed as frequency counts (n) and percentages (%). The chi-square test was applied to analyze categorical data. Statistical significance was defined as a *P*-value of less than 0.05.

## 3 Results

**Figure 1:**
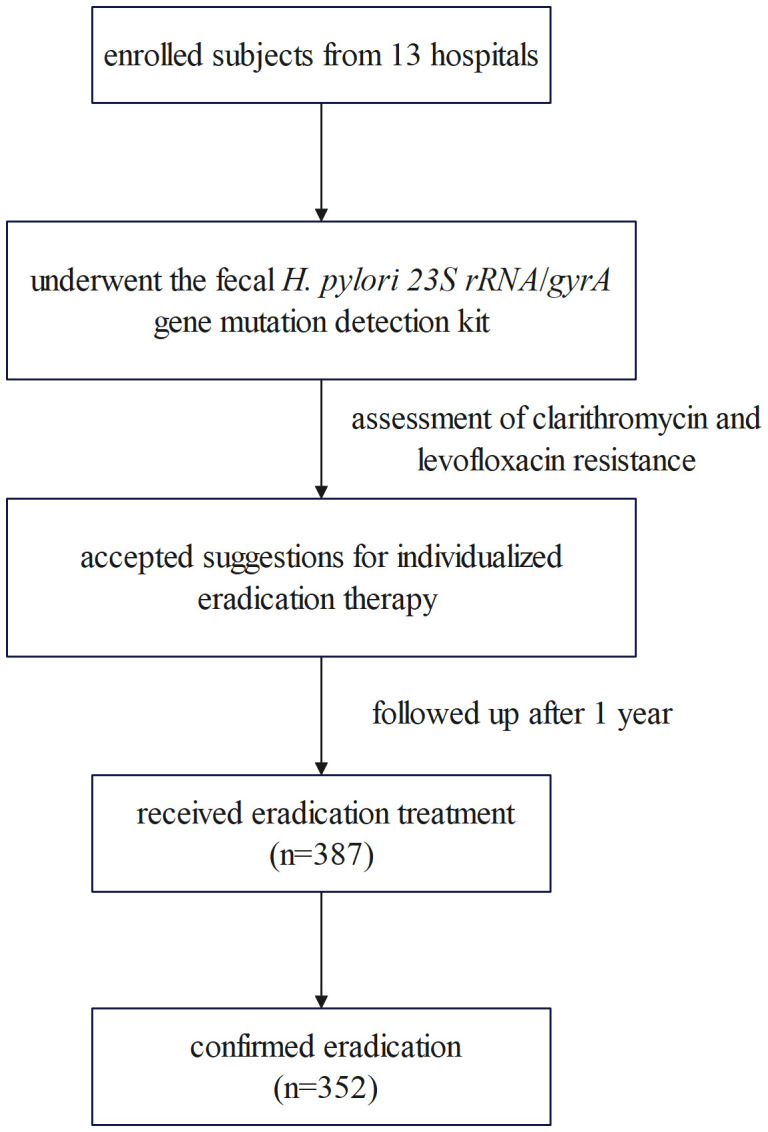
Research flowchart.

A total of 387 *H. pylori*-infected patients received eradication treatment and underwent a UBT across 13 hospitals. Among these, 352 patients successfully eradicated *H. pylori*, yielding an overall eradication rate of 91.0% (352/387, 95% CI: 87.6%-93.6%). The flowchart is depicted in **Figure 1**.

As detailed in **Table 1**, of the 387 patients enrolled in the study, 310 were administered a BQT, while 77 received a HDDT, with both treatment regimens lasting for a duration of 14 days. The eradication rates for BQT and HDDT were 90.0% (279/310, 95% CI: 86.1%-93.1%) and 94.8% (73/77, 95% CI: 87.2%-98.6%), respectively, while there was no statistical significance (*P*=0.188). Among the 310 patients treated with the BQT, only 3 used vonoprazan, a novel P-CAB, while the remaining 307 used the classic acid-suppressing drug—PPI. In the analysis of the BQT regimens, the inclusion of two different antibiotics did not significantly influence the eradication rates (*P*=0.208). The majority of patients, 142 in total, were treated with BQT that included amoxicillin and furazolidone, achieving an eradication rate of 93.0% (132/142, 95% CI: 87.4%-96.6%). Following this, 42.6% (132/310) of patients were administered the amoxicillin and clarithromycin combination, which led to 115 successful eradications, corresponding to an eradication rate of 87.1% (95% CI: 80.2%-92.3%). 22 and 12 patients received BQT containing amoxicillin and levofloxacin, and amoxicillin and doxycycline or tetracycline, respectively. The remaining 2 patients, due to a history of penicillin allergy, chose BQT with furazolidone and either doxycycline or tetracycline. The eradication rates were 81.8% (18/22, 95% CI: 59.7%-94.8%), 100.0% (12/12, 95% CI: 73.5%-100.0%), and 100.0% (2/2, 95% CI: 15.8%-100.0%), respectively.

**Table 1:** Comparison of eradication rates of different treatment regimens.

Group	Received therapy	Confirmed eradication	Eradication rate (95% CI)	χ^2^	*P* value
BQT	310	279	90.0% (86.1%-93.1%)	1.731	0.188
HDDT	77	73	94.8% (87.2%-98.6%)		
Group (BQT)	Received therapy	Confirmed eradication	Eradication rate (95% CI)	χ^2^	*P* value
AMX+CLA	132	115	87.1% (80.2%-92.3%)	5.381	0.208
AMX+LVX	22	18	81.8% (59.7%-94.8%)		
AMX+FZD	142	132	93.0% (87.4%-96.6%)		
AMX+DOX or TET	12	12	100.0% (73.5%-100.0%)		
FZD+DOX or TET	2	2	100.0% (15.8%-100.0%)		
Group (HDDT)	Received therapy	Confirmed eradication	Eradication rate (95% CI)	χ^2^	*P* value
ESO-based HDDT	14	12	85.7% (57.2%-98.2%)	1.058	0.304
VPZ-based HDDT	63	61	96.8% (89.0%-99.6%)		

There are no significant differences among various treatment groups.

Furthermore, the HDDT regimen was classified based on the type of acid-suppressing agent used: HDDT with vonoprazan and HDDT with esomeprazole. Although the eradication rate in the vonoprazan-based HDDT was higher at 96.8% (61/63, 95% CI: 89.0%-99.6%) compared to 85.7% (12/14, 95% CI: 57.2%-98.2%) in the esomeprazole-based HDDT, this difference was not statistically significant (*P*=0.304).

## 4 Discussion

To the best of our knowledge, this study is the first to evaluate the use of PCR-based detection of clarithromycin and levofloxacin resistance genotypes in fecal samples to guide individualized *H. pylori* eradication therapy in a real-world setting. In our study, after applying the results of this testing to clinical treatment, the overall eradication rate reached 90.8%, a success that was independent of the specific treatment regimen employed. The findings of this study provide robust evidence supporting the efficacy of using fecal PCR for the detection of *23S rRNA* and *gyrA* genes to guide individualized therapy, confirming the potential and value of this method for broader clinical application following further research.

According to the 2022 Chinese national guidelines (Zhou et al., 2022), the first-line *H. pylori* eradication treatment remains the classic BQT, and the HDDT is firstly recommended as an alternative first- and second-line treatment option for *H. pylori* -infected patients. The recommended course of treatment is 14 days. HDDT refers to the use of double the standard dose of PPI in combination with high-dose amoxicillin (≥ 3 g/d, TID or QID). In recent years, the novel HDDT that replaces PPI with P-CAB, represented by vonoprazan, combined with high-dose amoxicillin, has demonstrated ideal eradication rates in randomized controlled trials nationwide. The efficacy of P-CAB-based HDDT is non-inferior to that of BQT (Chen et al., 2024; Hu et al., 2023; Wang et al., 2023) and PPI-based HDDT (Su et al., 2023; Yan et al., 2024). Therefore, this study found in the real world that the majority of patients still use BQT, with no significant difference in eradication rates among groups using different antibiotic combinations guided by clarithromycin and levofloxacin susceptibility. A minority of other patients received PPI-based and P-CAB-based HDDT therapy, with comparable eradication rates in both groups. These treatment options are in line with China’s national conditions and clinical practices.

In our country, BQT remains the preferred treatment option for *H. pylori* eradication. However, due to the increasing problem of antibiotic resistance, its efficacy has not reached the expected ideal level. An analysis of 24 randomized controlled trials found that the average eradication rate of BQT was only 81.3% (Zhou et al., 2022), failing to exceed the satisfactory threshold of 90%. Currently, there are six antibiotics available for *H. pylori* eradication treatment in our country, including clarithromycin, levofloxacin, metronidazole, amoxicillin, furazolidone, and tetracycline. The resistance rates of the first three antibiotics are high, while those of the latter three remain relatively low (Zhong et al., 2021). Resistance to antibiotics largely determines the successful eradication. A meta-analysis (Zou et al., 2020) that comprehensively assessed the eradication situation of nearly 30,000 patients showed that compared with the antibiotic-sensitive group, the eradication rate for the clarithromycin-resistant group dropped to 57.29%, and for the levofloxacin-resistant group, it dropped to 75%. Despite a high resistance rate, increasing the dose may partly overcome the metronidazole resistance, whereas the resistance to clarithromycin and levofloxacin cannot be overcome by increasing the dose (Liou et al., 2022). Given their widespread application as initial treatment options, ascertaining the susceptibility of clarithromycin and levofloxacin is imperative prior to their clinical deployment.

The antimicrobial susceptibility test, as a classic method for assessing antibiotics resistance, is limited due to the need for specific culture conditions, extended culture times, and the requirement for gastric mucosal sampling through endoscopy. It is widely recognized that clarithromycin and levofloxacin resistance genotypes and phenotypes show good concordance (Zhong et al., 2021). Detecting resistance genotypes to these two antibiotics holds significant clinical value for guiding *H. pylori* eradication therapy. Two multicenter randomized controlled trials conducted in Taiwan demonstrated that treatment guided by molecular detection of the *23S rRNA* and *gyrA* genes is as effective as traditional culture-based susceptibility testing in first-line and third-line treatment of *H. pylori* infection, with eradication rates between 86% and 88% (Chen et al., 2023). A prospective clinical study by Han et al. (2023) also found that a 14-day susceptibility-guided BQT, informed by PCR detection of clarithromycin and levofloxacin resistance, achieved an eradication rate as high as 91.8%. The aforementioned findings provide robust interpretation and evidential support for the reliability of this study, underscoring the importance and potential clinical application value of genotype testing for clarithromycin and levofloxacin in the eradication treatment of *H. pylori*.

## 5 Conclusion

In conclusion, the findings of this study demonstrate that the fecal PCR-based detection of *23S rRNA* and *gyrA* gene mutations, utilized to inform personalized treatment strategies, can achieve an overall eradication rate exceeding 90%. This approach merits consideration for broader adoption in clinical settings due to its efficacy.

## 6 Limitations

While this real-world study offers pragmatic insights into clinical practice, several inherent limitations must be acknowledged. Firstly, as a real-world study, the absence of a control group precludes direct comparison with empirical treatment eradication rates, given the compromised comparability arising from heterogeneous participant selection criteria. Secondly, therapeutic regimens were not standardized and were subject to physician preferences or patient-specific factors (e.g., comorbidities, drug availability). This variability resulted in uneven subgroup distributions, with some treatment cohorts having insufficient sample sizes for robust statistical comparisons. Finally, the non-interventional design precluded systematic monitoring of medication adherence or adverse events, relying instead on patient self-reporting and sporadic clinical documentation. This unsupervised approach risks underestimating non-compliance rates and missing undocumented side effects, potentially biasing outcome assessments.

## Data Availability

The raw data supporting the conclusions of this article will be made available by the authors, without undue reservation.
