# Supplementary material for: Clinical evaluation of personalized Helicobacter pylori treatment guided by PCR detection from fecal samples: a real-world study
Source: Front Cell Infect Microbiol. 2025 Apr 28;15:1519804. doi: 10.3389/fcimb.2025.1519804 (PMC12066622; doi:10.3389/fcimb.2025.1519804)
Supplement: Supplementary file 1 [file Table1.docx]

**Supplementary Table 1 PCR primers and mutation sites to determine the clarithromycin and levofloxacin resistance**

| Genes | Primers | Mutation sites | Annotation |
| --- | --- | --- | --- |
| *23S rRNA* | Forward: 5’-GCATGAATGGCGTAACGAGAT-3’  Reverse: 5’-ATAAGAGCCAAAGCCCTTACTTCAAAG-3’ | A2143G andA2142G/C | Related to clarithromycin resistance |
| *gyrA* | Forward: 5’-GATCGTGGGTGATGTGATTGGTA-3’ | N87I/K and D91N/Y/G | Related to levofloxacin resistance |
|  | Reverse: 5’-AAAATCTTGCGCCATTCTCACTA-3’ |  |  |
| ACTB | Forward: 5’-CCATCCTGCGTCTGGACCT-3’ | / | Internal reference gene |
|  | Reverse: 5’-CCGTGGTGGTGAAGCTGTAG-3’ |  |  |

**Supplementary Table 2 PCR reaction conditions**

| Stage | Temperature | Time | Number of amplification cycles |
| --- | --- | --- | --- |
| Pre-denaturation | 94℃ | 1 min | 1 |
| Extension | 94℃ | 15 s | 55 |
|  | 60℃ | 30 s |  |
| Melting curve | 94℃ | 30 s | 1 |
|  | 45℃ | 1 min |  |
|  | 45℃-75℃ | / |  |
|  | 75℃ | 30 s | 1 |
| Annealing | 25℃ | 15 s |  |
